# Impact of the severity of restrictive spirometric pattern on nutrition, physical activity, and quality of life: results from a nationally representative database

**DOI:** 10.1038/s41598-020-76777-w

**Published:** 2020-11-12

**Authors:** Sung Jun Chung, Hwan Il Kim, Bumhee Yang, Taehee Kim, Yun Su Sim, Hyung Koo Kang, Sang-Heon Kim, Ho Joo Yoon, Hayoung Choi, Hyun Lee

**Affiliations:** 1 Division of Pulmonary Medicine and Allergy, Department of Internal Medicine, Hanyang University College of Medicine, Seoul, South Korea; 2 Division of Pulmonary, Allergy, and Critical Care Medicine, Department of Internal Medicine, Hallym University Sacred Heart Hospital, Anyang, South Korea; 3 Division of Pulmonary and Critical Care Medicine, Department of Internal Medicine, Chungbuk National University Hospital, Cheongju, South Korea; 4 Division of Pulmonary, Allergy, and Critical Care Medicine, Department of Internal Medicine, Hallym University Kangnam Sacred Heart Hospital, Seoul, South Korea; 5 Lung Research Institute, Hallym University College of Medicine, Chuncheon, South Korea; 6 Division of Pulmonary and Critical Care Medicine, Department of Internal Medicine, Ilsan Paik Hospital, Inje University College of Medicine, Goyang, South Korea

**Keywords:** Respiratory tract diseases, Respiration, Quality of life

## Abstract

The general disease burden associated with the restrictive spirometric pattern (RSP) is substantial. However, the impact of RSP by its severity on general health problems and quality of life has not been well elucidated. This study aimed to analyse nutrition, physical activity, and quality of life in subjects who participated in the Korea National Health and Nutrition Examination Survey 2007–2016 according to severity of RSP. Participants were classified as subjects with normal spirometry, those with mild-to-moderate RSP, and those with severe RSP. Poor quality of life was defined as 25th percentile value on the EuroQoL five dimensions (Eq5D) questionnaire index, i.e., 0.90. This study included 23,615 subjects composed of 20,742 with normal spirometry, 2758 with mild-to-moderate RSP, and 115 with severe RSP. The subjects with severe RSP were more likely to have attained lower education levels, had a lower total caloric intake, had less physical activity, had experienced a higher prevalence of comorbidities, and poorer quality of life than those with normal spirometry (*P* < 0.001 for all). In multivariable analysis, subjects with a mild-to-moderate RSP and severe RSP were more likely to show decreased total calories (coefficient for change in calorie = − 56.6 kcal and − 286.7 kcal, respectively) than those with normal spirometry; subjects with mild-to-moderate RSP and those with severe RSP were 1.26 times and 1.96 times more likely, respectively, to have a poorer quality of life than those with normal spirometry. Additionally, subjects with mild-to-moderate RSP and those with severe RSP were 0.84 times and 0.36 times less likely, respectively, to have high-intensity physical activity than those with normal spirometry in univariable analysis. The trends of a poorer quality of life and physical activity were only significant in the male subgroups. In conclusion, our study revealed that the severity of general health problems and quality of life reductions are correlated with the severity of RSP, especially in males.

## Introduction

The restrictive spirometric pattern (RSP) is characterised by a matched deficit in forced expiratory volume in 1 s (FEV_1_) and forced vital capacity (FVC). The RSP is characterised by decreased FEV_1_ and FVC, and a preserved FEV_1_/FVC ratio^1^. Generally, the RSP has been identified in patients with interstitial lung diseases, pleural effusions, chest wall diseases, neuromuscular diseases, and diaphragmatic disorder^1,2^. However, recent studies have shown that RSP can also be observed in the aging population and in subjects with extra-thoracic conditions including diabetes mellitus, heart failure, metabolic syndrome, or obesity^2–5^. Thus, the RSP is relatively prevalent, 7–13%, in the general population^6^.

The impact of RSP on general health conditions is substantial. Recent studies evaluating participants in the US National Health and Nutritional Examination Survey (NHANES) showed that participants with RSP were more likely to report functional impairment and fair/poor health than were those with no lung disease and normal lung function^7,8^. RSP is also related to increased respiratory symptoms^6^, multiple comorbidities^9^, poor quality of life^10^, and mortality^11^.

However, despite a significant association between RSP and those general health problems and quality of life, whether or not those health problems and quality of life worsen proportionally to the severity of RSP has not been well elucidated. Hence, this study aimed to investigate the impact of the severity of RSP on physical activity, nutrition, and quality of life using a national database.

## Methods

### Study population

The Korea NHANES is a cross-sectional survey of the non-institutionalized South Korean population conducted by the Korean Ministry of Health and Welfare using a stratified, multistage clustered probability sampling design. Sampling units were defined on the basis of household registries, including geographic area, sex, and age groups^12^. All data from this survey are publicly available through the Korea NHANES website^13^.

We constructed a cohort of subjects with available pulmonary function test (PFT) results from the Korea NHANES using data obtained from January 2007 to December 2016. Of the subjects with available PFT results, this study investigated subjects with normal or restrictive spirometric results after excluding those with obstructive spirometric results. The 2007–2016 Korea NHANES study protocols were approved by the Institutional Review Boards of the Korean Centers for Disease Control and Prevention. Written informed consent was obtained from all participants.

### Measurements

The Korea NHANES included a standardized questionnaire administered at home by a trained interviewer and a detailed physical examination administered at a mobile examination centre. All methods were carried out under the approved guidelines and regulations. Spirometry was performed according to the recommendations of the American Thoracic Society/European Respiratory Society^14^. Absolute values of FEV_1_ and FVC were obtained, and the percentage of predicted values (% predicted) for FEV_1_ and FVC were calculated using the reference equation obtained on analysis of a representative Korean sample^15^.

According to spirometric results, the study population was classified into normal spirometry, mild-to-moderate RSP, and severe RSP. Normal spirometry was defined as pre-bronchodilator FEV_1_/FVC ≥ 0.70 and FVC ≥ 80% predicted, and the obstructive spirometric pattern was defined as pre-bronchodilator FEV_1_/FVC < 0.70 irrespective of FVC values^16^. RSP was defined as pre-bronchodilator FEV_1_/FVC ≥ 0.70 and FVC < 80% predicted^1^ and was subdivided into mild-to-moderate RSP (60% predicted ≤ FVC < 80% predicted) and severe RSP (FVC < 60% predicted)^17^.

Demographic information, education, smoking history, monthly family income, medical history, and medication use were determined by self-report. Comorbidities included hypertension, dyslipidaemia, diabetes mellitus, cardiovascular disease, asthma, osteoporosis, osteoarthritis, and malignancy; these were based on self-reports of physician diagnosis and laboratory data^18^. Regarding physical activity, moderate-intensity physical activity was defined as “activity that causes small increases in breathing or heart rate, such as brisk walking or carrying light loads for at least 10 min continuously”; high-intensity physical activity was defined as “activity that causes large increases in breathing or heart rate such as carrying or lifting heavy loads, digging, or construction work for at least 10 min continuously”^13^.

The EuroQoL five dimensions questionnaire (EQ-5D) index values were obtained. These values included five arms (mobility, self-care, usual activities, pain/discomfort, and anxiety/depression). EQ-5D index values range between 0 (worst imaginable health status) and 1 (best imaginable health state). EQ-5D index score was defined as the quality of life index, which was calculated by the quality of life estimation published by the Korean Centers for Disease Control. We used the 25th percentile of EQ-5Q index values as a cut-off value^19^.

### Statistical analysis

All statistical analyses were performed using NHANES weights and svy (survey) commands in STATA (Release 13.1; StataCorp LP, College Station, TX, USA) to account for the complex multistage probability sampling design. Pulmonary function tested subsample weights were used in all analyses to account for the additional stage of sampling^20^. The exposures were RSP and its severity, and the outcomes were nutrition, physical activity, and quality of life.

The main outcomes of this study—nutrition, physical activity, and quality of life—were assessed as follows: nutrition was assessed by total calorie intake per day (kcal), physical activity was assessed by the rate of days doing high-intensity physical activity during the last week, and quality of life was assessed as low quality versus high quality using the cut-off value (0.9) of the EQ-5D index.

Univariable and multivariable linear regression analyses were performed to assess the impact of RSP and its severity on total calories, and univariable and multivariable logistic regression analyses were performed to assess the impact of RSP and its severity on high-intensity physical activity, EQ-5D values, and EQ-5D components. Multivariable analysis was adjusted for age (≥ 65 years versus < 65 years), sex, smoking status (never-smokers versus current- or ex-smokers), body mass index (BMI) (BMI ≥ 25 kg/m^2^ versus 18.5 kg/m^2^ ≤ BMI < 25 kg/m^2^ versus BMI < 18 kg/m^2^), education level (high school or less versus college or above), family income (upper half versus lower half), and the number of comorbidities (one or less versus two or more). The potential confounding factors adjusted for multivariable analysis included clinically important variables or statistically significant variables in the univariable analysis. To examine whether sex was an effect modifier, *P* for interaction was obtained from the likelihood ratio tests for interaction with normal spirometry/mild-to-moderate RSP/severe RSP and sex. Additionally, we presented the linear and logistic regression analyses in the overall, male, and female populations, respectively.

## Results

### Clinical characteristics of the study population

**Figure 1:**
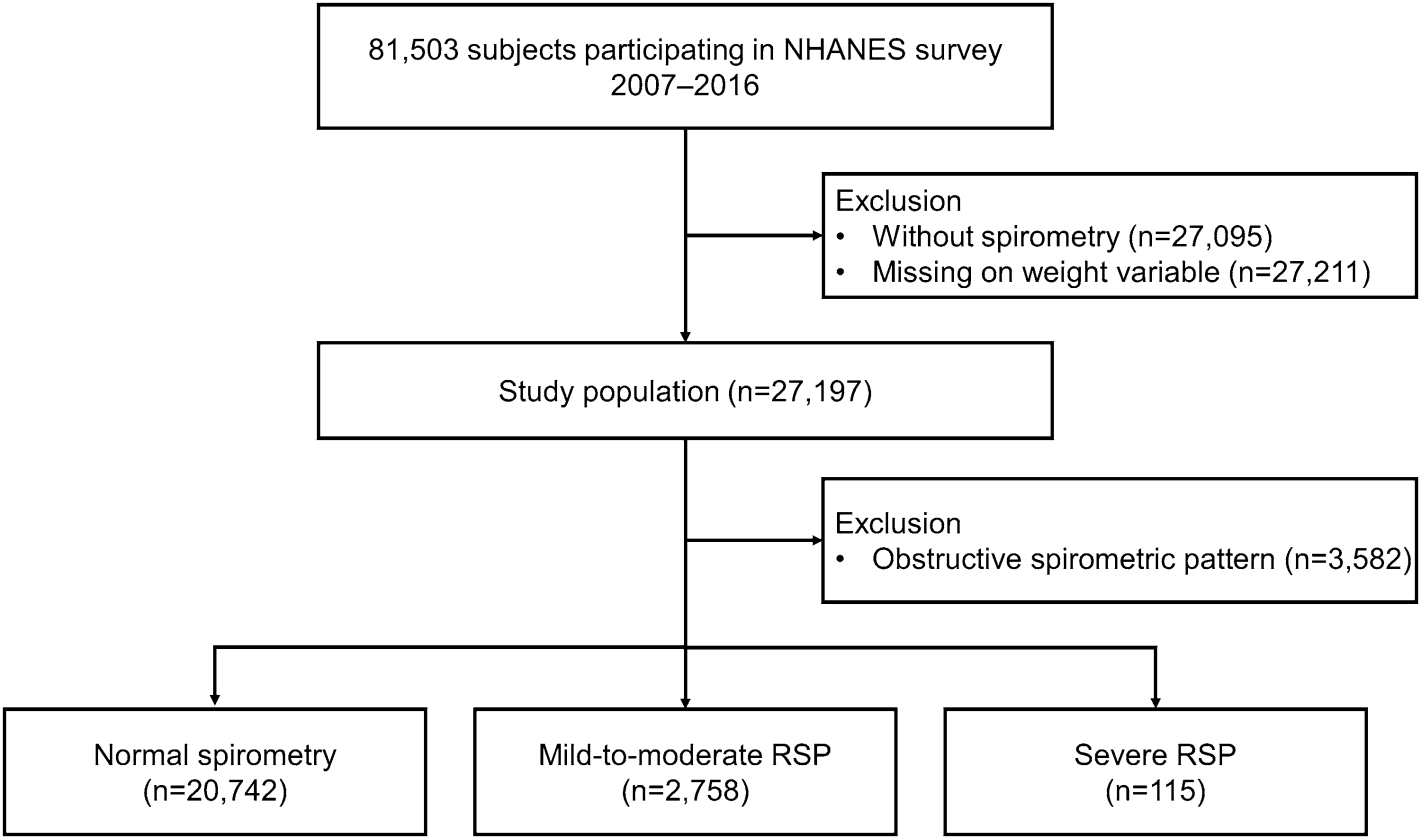
Flow chart of the study population. *NHANES* National Health and Nutritional Examination Survey, *RSP* restrictive spirometric pattern.

A total of 81,503 subjects were identified from the Korea NHANES database between 2007 and 2016. Subjects who had missing weight variables (n = 27,211) and PFT (n = 27,095) were excluded. Among the subjects, after excluding 3582 with an obstructive spirometric pattern, we enrolled 23,615 subjects composed of 20,742 with normal spirometry, 2758 with mild-to-moderate RSP, and 115 with severe RSP (Fig. 1).

Subjects with RSP were older than those with normal spirometry (49.7 years in normal, 55.4 years in mild-to-moderate RSP, and 64.1 years in severe RSP; *P* < 0.001). The ratio of male sex was significantly higher in subjects with RSP (50.6% in mild-to-moderate RSP and 54.4% in severe RSP) than those with normal spirometry (*P* < 0.001). Subjects with RSP showed larger waist circumference than those with normal spirometry (82.5 cm in normal, 86.5 cm in mild-to-moderate RSP, and 85.9 cm in severe RSP; *P* < 0.001). In addition, the subjects with a severe RSP tended to have lower education grades (high school or less) (93.2%) compared to those with other spirometric patterns (70.8% with normal spirometry and 76.4% with mild-to-moderate RSP) (*P* < 0.001). Regarding comorbidities, subjects with a severe RSP showed a significantly higher prevalence of diabetes mellitus, hypertension, cardiovascular diseases, osteoporosis, and osteoarthritis (*P* < 0.001 for all) (Table 1).

**Table 1:** Baseline characteristics of the study population according to spirometric patterns.

	Total (N = 23,615)	Normal spirometry (n = 20,742)	Mild-to-moderate RSP (n = 2758)	Severe RSP (n = 115)	*P*
Age, years	50.3 (0.2)	49.7 (0.2)	55.4 (0.4)	64.1 (2.5)	< 0.001
Sex, male	45.4 (0.4)	44.7 (0.4)	50.6 (1.2)	54.4 (5.6)	< 0.001
BMI, kg/m^2^	24.3 (0.0)	24.2 (0.0)	25.3 (0.1)	24.6 (0.5)	< 0.001
Waist circumference, cm	83.0 (0.1)	82.5 (0.1)	86.5 (0.3)	85.9 (1.4)	< 0.001
**Smoking status**					0.022
Current smoker	27.8 (0.4)	27.6 (0.4)	29.3 (1.2)	26.3 (5.2)	
Ex-smoker	12.7 (0.3)	12.5 (0.3)	14.5 (0.8)	16.3 (4.3)	
Never-smoker	59.5 (0.4)	59.9 (0.4)	56.2 (1.3)	57.4 (5.7)	
**Education level**					< 0.001
High school or less	71.5 (0.5)	70.8 (0.6)	76.4 (1.2)	93.2 (2.2)	
College or above	28.5 (0.5)	29.2 (0.6)	23.6 (1.2)	6.8 (2.2)	
**Marital status**					0.159
Married or living together	80.1 (0.4)	80.3 (0.5)	79.0 (1.1)	72.5 (5.7)	
Single/separated/divorced/widowed	19.9 (0.4)	19.7 (0.5)	21.0 (1.1)	27.5 (5.7)	
**Family income**^a^					< 0.001
Low	40.8 (0.6)	39.8 (0.6)	48.1 (1.3)	62.9 (5.9)	
High	59.2 (0.6)	60.2 (0.6)	51.9 (1.3)	37.1 (5.9)	
**Comorbidities**					
Diabetes mellitus	10.7 (0.2)	9.4 (0.2)	20.5 (0.9)	30.2 (5.0)	< 0.001
Hypertension	32.9 (0.4)	30.9 (0.4)	48.3 (1.2)	55.4 (5.9)	< 0.001
Dyslipidemia	41.1 (0.4)	40.1 (0.4)	49.1 (1.2)	37.2 (5.1)	< 0.001
Cardiovascular diseases	3.2 (0.1)	2.8 (0.1)	6.3 (0.5)	14.9 (3.9)	< 0.001
Asthma	1.9 (0.1)	1.8 (0.1)	2.9 (0.3)	3.8 (1.7)	< 0.001
Osteoporosis	17.3 (0.7)	16.3 (0.7)	24.4 (1.9)	27.0 (7.8)	< 0.001
Osteoarthritis	27.9 (0.5)	26.9 (0.6)	34.7 (1.5)	40.2 (7.0)	< 0.001
Malignancy	3.6 (0.1)	3.4 (0.1)	4.8 (0.5)	10.8 (3.3)	< 0.001
**Pulmonary function test**					
FVC, L	3.63 (0.0)	3.72 (0.0)	2.88 (0.0)	1.95 (0.0)	< 0.001
FVC, %predicted	93.2 (0.1)	95.7 (0.1)	74.5 (0.1)	54.1 (0.5)	< 0.001
FEV_1_, L	2.91 (0.0)	2.99 (0.0)	2.32 (0.0)	1.55 (0.0)	< 0.001
FEV_1_, %predicted	93.9 (0.1)	96.0 (0.1)	78.0 (0.2)	59.1 (0.8)	< 0.001
FEV_1_/FVC	0.80 (0.0)	0.80 (0.0)	0.81 (0.0)	0.79 (0.0)	0.068

Values are presented as mean (standard deviation) for age, BMI, waist circumference, and pulmonary function test and % (standard deviation) for other variables.

*RSP* restrictive spirometric pattern, *BMI* body mass index, *FVC* forced vital capacity, *FEV*_*1*_ forced expiratory volume in 1 s.

^a^Family income levels were classified as upper half and lower half.

### Nutrition, physical activity, and quality of life according to spirometric results

Regarding nutritional uptake, total calories were significantly lower in subjects with a severe RSP (1661.1 ± 76.4 kcal) compared to those with other spirometric patterns (2001.4 ± 8.7 kcal in normal and 1935.9 ± 22.6 kcal in mild-to-moderate RSP) (*P* < 0.001). In terms of physical activity, subjects with a severe RSP showed a significantly lower ratio of high-intensity physical activity (6.8% vs. 16.8% in normal and 14.5% in mild-to-moderate RSP; *P* = 0.018) and fewer days of high-intensity physical activity (1.6 days vs. 2.5 days in normal and 2.8 days in mild-to-moderate RSP; *P* < 0.001) compared to those with other spirometric patterns.

In terms of the EQ-5D index, which represents the quality of life, the severe RSP group had a significantly lower value (0.88) compared to other groups according to spirometric results (0.95 in normal and 0.92 in mild-to-moderate RSP) (*P* < 0.001). The proportion of subjects with EQ-5D values less than 0.9 was significantly higher in the severe RSP group than the other groups (40.5% vs. 16.6% in normal and 24.6% in mild-to-moderate RSP; *P* < 0.001) (Table 2).

**Table 2:** Nutrition, physical activity, and quality of life according to spirometric patterns.

	Total (N = 23,615)	Normal spirometry (n = 20,742)	Mild-to-moderate RSP (n = 2758)	Severe RSP (n = 115)	*P*
**Total calories, kcal**	1993.0 (8.2)	2001.4 (8.7)	1935.9 (22.6)	1661.1 (76.4)	< 0.001
Carbohydrate, g	317.0 (1.2)	317.4 (1.3)	315.4 (3.2)	283.6 (11.8)	0.016
Protein, g	70.2 (0.4)	70.6 (0.4)	67.3 (0.9)	59.8 (7.8)	0.001
Daily fat intake, g	39.6 (0.3)	40.1 (0.3)	36.4 (0.8)	26.8 (2.5)	< 0.001
Fatty acid intake, g					
Saturated fat intake, g	11.6 (0.1)	11.7 (0.2)	10.7 (0.4)	8.4 (1.3)	0.004
Unsaturated fat intake, g	23.5 (0.3)	23.7 (0.3)	21.8 (0.8)	15.8 (2.0)	< 0.001
Water, g	1064.4 (6.7)	1076.9 (7.1)	971.3 (16.9)	827.2 (51.3)	< 0.001
**Physical activity**					
High-intensity physical activity	16.5 (0.4)	16.8 (0.4)	14.5 (1.0)	6.8 (3.1)	0.018
Days of high-intensity physical activity per week	2.5 (0.1)	2.5 (0.1)	2.8 (0.3)	1.6 (0.2)	< 0.001
Moderate-intensity physical activity	11.1 (0.4)	11.4 (0.4)	9.0 (0.8)	11.5 (4.4)	0.036
Days of moderate-intensity physical activity per week	2.9 (0.1)	2.8 (0.1)	3.2 (0.3)	2.5 (0.3)	0.3421
**EQ-5D index values < 0.9**	17.5 (0.3)	16.6 (0.3)	24.6 (1.0)	40.5 (5.4)	< 0.001

Values are presented as % (standard deviation) for high-intensity physical activity, moderate-intensity physical activity and EQ-5D index values < 0.9, and mean (standard deviation) for other variables.

*RSP* restrictive spirometric pattern, *EQ-5D* EuroQoL five-dimensions questionnaire.

### Impact of spirometric patterns on nutrition, physical activity, and quality of life according to spirometric patterns

Regardless of adjustment for covariables, subjects with a mild-to-moderate RSP were more likely to show decreased total calories (adjusted coefficient for change in calorie = − 56.6 kcal, 95% confidence interval [CI] − 101.9 to − 11.4 kcal) compared with those with normal spirometry. Furthermore, subjects with severe RSP were even more likely to show decreased total calories (adjusted coefficient for change in calorie = − 286.7 kcal, 95% CI − 431.7 to − 141.6 kcal) compared with those with normal spirometry. The impact of RSP and its severity on nutrition were also observed in the male and female subgroups (Table 3).

**Table 3:** Unadjusted and adjusted odds ratio for total calories, physical activity, and EQ-5D values according to spirometric patterns.

			Normal spirometry (n = 20,742)	Mild-to-moderate RSP (n = 2758)	Severe RSP (n = 115)
Total calories, kcal	Overall	Univariable	Reference	− 65.6 (− 112.8 to − 18.3)	− 340.3 (− 491.3 to − 189.3)
		Multivariable	Reference	− 56.6 (− 101.9 to − 11.4)	− 286.7 (− 431.7 to − 141.6)
	Male	Univariable	Reference	− 163.1(− 240.6 to − 85.5)	− 568.2 (− 778.6 to − 357.9)
		Multivariable	Reference	− 103.4 (− 184.3 to − 22.5)	− 348.3 (− 554.6 to − 142.1)
	Female	Univariable	Reference	− 53.1 (− 94.8 to − 11.4)	− 225.5 (− 397.7 to − 53.2)
		Multivariable	Reference	− 3.5 (− 45.6 to 38.6)	− 156.6 (− 349.6 to 36.4)
			*P*_*Interaction*_	< 0.001	< 0.001
High-intensity physical activity	Overall	Univariable	Reference	0.84 (0.71 to 0.99)	0.36 (0.14 to 0.95)
		Multivariable	Reference	0.90 (0.75 to 1.08)	0.45 (0.17 to 1.21)
	Male	Univariable	Reference	0.88 (0.69 to 1.12)	0.09 (0.01 to 0.67)
		Multivariable	Reference	0.92 (0.72 to 1.19)	0.12 (0.02 to 0.88)
	Female	Univariable	Reference	0.74 (0.57 to 0.96)	0.85 (0.27 to 2.71)
		Multivariable	Reference	0.83 (0.64 to 1.08)	1.07 (0.32 to 3.66)
			*P*_*Interaction*_	0.290	0.019
EQ-5D values < 0.9	Overall	Univariable	Reference	1.64 (1.46 to 1.84)	3.43 (2.20 to 5.33)
		Multivariable	Reference	1.26 (1.10 to 1.43)	1.96 (1.22 to 3.17)
	Male	Univariable	Reference	2.27 (1.88 to 2.76)	7.07 (3.93 to 12.72)
		Multivariable	Reference	1.68 (1.36 to 2.07)	3.49 (1.89 to 6.41)
	Female	Univariable	Reference	1.54 (1.34 to 1.76)	2.27 (1.15 to 4.49)
		Multivariable	Reference	1.07 (0.91 to 1.26)	1.13 (0.60 to 2.12)
			*P*_*Interaction*_	< 0.001	< 0.001

Values are presented as coefficient (95% confidence interval) in total calories and odds ratio (95% confidence interval) in high-intensity physical activity and EQ-5D values. Multivariable analysis was adjusted for age (≥ 65 years versus < 65 years), sex, smoking status (never smokers versus current- or ex-smokers), BMI (BMI ≥ 25 kg/m^2^ versus 18.5 kg/m^2^ ≤ BMI < 25 kg/m^2^ versus BMI < 18 kg/m^2^), education level (high school or less versus college or above), family income (upper half versus lower half), and the number of comorbidities (one or less versus two or more). *P* for interaction (*P*_*Interaction*_) was obtained from likelihood ratio tests for interaction with normal spirometry/mild-to-moderate RSP/severe RSP and sex.

*RSP* restrictive spirometric pattern, *EQ-5D* EuroQoL five-dimensions questionnaire, *BMI* body mass index.

Subjects with mild-to-moderate RSP (unadjusted odds ratio [OR] 0.84, 95% CI 0.71–0.99) and those with severe RSP (unadjusted OR 0.36, 95% CI 0.14–0.95) were less likely to show high-intensity physical activity compared with those with normal spirometry. However, the impact of RSP and its severity on high-intensity physical activity did not persist after adjusting for covariables (Table 3).

Subjects with mild-to-moderate RSP were more likely to have impaired quality of life, defined as EQ-5D index less than 0.9, compared to those with normal spirometry (unadjusted OR 1.64, 95% CI 1.46–1.84; adjusted OR 1.26, 95% CI 1.10–1.43). Furthermore, subjects with severe RSP were even more likely to have impaired quality of life compared to those with normal spirometry (unadjusted OR 3.43, 95% CI 2.20–5.33; adjusted OR 1.96, 95% CI 1.22–3.17). The trends were also observed in both univariable and multivariable analyses in males; however, it was only observed in univariable analysis in females (*P* for interactions < 0.001) (Table 3). Supplementary Table S1 is provided to assess the impact of the spirometric pattern of the EQ-5D components. Specifically, even after adjustment for covariables, subjects with severe RSP were more likely to have impaired quality of life associated with the following arms: self-care arm (adjusted OR 2.64, 95% CI 1.37–5.10) and usual activities arm (adjusted OR 2.15, 95% CI 1.28–3.61).

## Discussion

To the best of our knowledge, this is the first study to evaluate general health status, including nutrition, physical activity, and quality of life, in subjects with RSP based on its severity. In this study, subjects with RSP were more likely to show older age, larger waist circumference, lower education level attainment, less physical activity, and more comorbidities compared with those with normal spirometry. Interestingly, the differences were more profound in subjects with severe RSP. Subjects with severe RSP attained even lower education levels, had lower total caloric intake, were less physically active, and had a higher prevalence of comorbidities (e.g., diabetes mellitus, hypertension, cardiovascular disease, asthma, osteoporosis, osteoarthritis, and malignancy) than those with mild-to-moderate RSP. Furthermore, especially in males, the severe RSP group was more likely to have lower total calorie intake, lower high-intensity physical activity, and impaired quality of life measured by the EQ-5D index compared with subjects with normal spirometry.

There is accumulating evidence, including our data, that the RSP is related to smoking, aging, reduced physical activity, poorer quality of life, and many medical ailments. These ailments include obesity, diabetes mellitus, and cardiovascular diseases^6,7,21^. In agreement with previous reports^1,22^, the rates of ex- or current smokers were 78.7% in males and 7.5% in females in this study, which suggests an association between RSP and smoking. However, there have been no studies that evaluated the relationship between these conditions and the severity of RSP. Our study revealed that the prevalence of those medical ailments differed significantly according to the severity of RSP. In this study, age, being male, physical inactivity, and the prevalence of comorbid profiles, such as diabetes mellitus, hypertension, cardiovascular diseases, osteoporosis, osteoarthritis, and malignancy, increased proportionally to the RSP severity. Accordingly, our results suggest that clinicians need to focus on RSP severity in addition to its presence or absence^23^.

The subjects with RSP demonstrated poorer quality of life that was proportional to the severity of RSP. In agreement with our study findings, Guerra and colleagues, using two large population-based cohorts, showed that the association between RSP and deficits in the physical component of quality of life was partly independent of the presence of respiratory symptoms^10^. Our study added value to that previous study in that we discovered that the subjects with RSP were more likely to have difficulties in mobility, self-care, and pain/discomfort in addition to usual activities. Furthermore, these tendencies were more significant in subjects with severe RSP than those with mild-to-moderate RSP.

Another important finding of our study was the linkage between daily diet and BMI and RSP. Interestingly, despite a close relationship between BMI and RSP^24^, there have been no studies that evaluated the relationship between daily diet and RSP. Compared with subjects with normal spirometry, the RSP group had lower total caloric, carbohydrate, protein, fat, and fatty acid intake despite having higher BMI. The reason for our results may be that the RSP group was more likely to be older and have more comorbidities leading to physical inactivity. Physical inactivity might have influenced the subjects with RSP to display low caloric intake or vice versa. Although the exact mechanism cannot be deduced from this study result, our study provided informative data that linked RSP to diet. Emphasis needs to be placed on the finding that subjects with RSP had a lower caloric, carbohydrate, protein, fat, and fatty acid intake despite having higher BMI.

Interestingly, the associations between RSP severity and physical activity (or quality of life) were only significant in the male subgroup. In agreement with our findings, subjects with RSP from two large population-based cohorts revealed higher rates of males compared to those with normal spirometry or obstructive pattern: 53% in the European Respiratory Health Survey (ECRHS 2) and 56% in Swiss Cohort on Air Pollution and Lung and Heart Disease in Adults (SAPALDIA 2)^10^. Despite the higher male ratio in RSP patterns of the ECRHS 2 and SAPALDIA 2 studies, the studies did not investigate the according to sex. Accordingly, this study has the strength to comprehensively assess the impact of RSP and its severity on nutrition, physical activity, and quality of life based on sex. From this study’s findings, male sex might affect general health problems that were considered to be related to RSP. However, this study cannot explain the underlying mechanism of this phenomenon. Future studies are warranted to clarify this issue.

Understanding the mechanism of a disease condition and determining appropriate treatment based on the pathophysiology remain essential strategies to control the disease. Therefore, our study results, with the findings of previous studies^25–28^, may indicate that the health problems related to RSP might be reduced by the controlling of some of the modifiable factors such as low physical activity and obesity. However, as our study is cross-sectional, we could not evaluate causal inference. Thus, a well-designed prospective study is needed to evaluate the causal inference of RSP and these factors and to investigate whether RSP can be improved by the intervention of some modifiable factors.

One important advantage of our study is in the representativeness of the general population; the nationwide database population cohort used represented the Korean population^29^. Previous studies using ECRHS, SAPALDIA, and COPDgene cohorts elucidated important health problems associated with RSP^10,20^. However, there may have been a selection bias due to the nature of cohort studies. Our study also has several limitations. First, due to the nature of the cross-sectional study design, reverse causation might limit drawing firm conclusions. Although there was a significant relationship between low caloric intake (or physical activity) and severity of RSP, subjects with severe RSP might eat less and do less physical activity. Thus, longitudinal studies are warranted to verify the findings of this study. Second, this study evaluated the representative Korean population; this might limit our ability to generalize our findings because of the possibility of race and ethnicity influences. Third, there could be information bias since demographic information, education, smoking history, monthly family income, and medical history were self-reported. Fourth, we did not have post-bronchodilator spirometric results. Therefore, some patients might have been misclassified due to using pre-bronchodilator spirometry.

In conclusion, subjects with RSP showed older age, larger waist circumference, attainment of lower education levels, less physical activity, and different comorbid profiles compared with those with normal spirometry. Furthermore, subjects with severe RSP had a lower total caloric intake, a lower rate of moderate- and high-intensity physical activity, a higher prevalence of comorbidities (diabetes mellitus, hypertension, cardiovascular disease, asthma, osteoporosis, osteoarthritis, and malignancy), and reduced quality of life compared to those with mild-to-moderate RSP. The impact of RSP and its severity on nutrition, physical activity, and quality of life were more profound, especially in males. Thus, understanding the characteristics of subjects with RSP as well as the difference according to the severity of RSP is essential for modifying risk factors of developing RSP and appropriately managing those with RSP.

## Supplementary information

Supplementary Table S1.

## Data Availability

All data extracted in this study are included in the current article.
