# Supplementary material for: Impact of the severity of restrictive spirometric pattern on nutrition, physical activity, and quality of life: results from a nationally representative database
Source: Sci Rep. 2020 Nov 12;10:19672. doi: 10.1038/s41598-020-76777-w (PMC7661506; doi:10.1038/s41598-020-76777-w)
Supplement: Supplementary file 1 — Supplementary Table S1. [file 41598_2020_76777_MOESM1_ESM.docx]

**Supplementary Information:** **Impact of the severity of restrictive spirometric pattern on nutrition, physical activity, and quality of life: results from a nationally representative database**

Sung Jun Chung^1^, Hwan Il Kim^2^, Bumhee Yang^3^, Taehee Kim^4,5^, Yun Su Sim^4,5^, Hyung Koo Kang^6^, Sang-Heon Kim^1^, Ho Joo Yoon^1^, Hayoung Choi^4,5^, Hyun Lee^1^

^1^Division of Pulmonary Medicine and Allergy, Department of Internal Medicine, Hanyang University College of Medicine, Seoul, Korea. ^2^Division of Pulmonary, Allergy, and Critical Care Medicine, Department of Internal Medicine, Hallym University Sacred Heart Hospital, Anyang, Korea. ^3^Division of Pulmonary and Critical Care Medicine, Department of Medicine, Chungbuk National University Hospital, Cheongju, Korea. ^4^Division of Pulmonary, Allergy, and Critical Care Medicine, Department of Internal Medicine, Hallym University Kangnam Sacred Heart Hospital, Seoul, Korea. ^5^Lung Research Institute, Hallym University College of Medicine, Chuncheon, Korea. ^6^Division of Pulmonary and Critical Care Medicine, Department of Internal Medicine, Ilsan Paik Hospital, Inje University College of Medicine, Goyang, Korea.

**Supplementary Table S1.** Unadjusted and adjusted odds ratio for EQ-5D components according to spirometric patterns

|  | |  | Normal spirometry (n = 20,742) | Mild-to-moderate RSP (n = 2,758) | Severe RSP (n = 115) |
| --- | --- | --- | --- | --- | --- |
| Mobility | Overall | Univariable | reference | 1.79 (1.58–2.03) | 3.00 (1.90–4.73) |
|  |  | Multivariable | reference | 1.26 (1.10–1.46) | 1.45 (0.86–2.42) |
|  | Male | Univariable | reference | 2.60 (2.10–3.20) | 5.99 (3.19–11.24) |
|  |  | Multivariable | reference | 1.82 (1.44–2.30) | 2.68 (1.37–5.25) |
|  | Female | Univariable | reference | 1.65 (1.41–1.92) | 2.13 (1.06–4.27) |
|  |  | Multivariable | reference | 1.05 (0.88–.125) | 0.89 (0.44–1.80) |
|  |  |  | *P _Interaction_* | <0.001 | <0.001 |
| Self-care | Overall | Univariable | reference | 1.99 (1.61–2.46) | 4.97 (2.77–8.92) |
|  |  | Multivariable | reference | 1.37 (1.09–1.72) | 2.64 (1.37–5.10) |
|  | Male | Univariable | reference | 3.09 (2.17–4.41) | 13.98 (6.84–28.59) |
|  |  | Multivariable | reference | 2.07 (1.38–3.10) | 5.94 (2.58–13.65) |
|  | Female | Univariable | reference | 1.68 (1.29–2.20) | 1.61 (0.58–4.48) |
|  |  | Multivariable | reference | 1.09 (0.83–1.44) | 0.92 (0.31–2.71) |
|  |  |  | *P _Interaction_* | <0.001 | <0.001 |
| Usual activities | Overall | Univariable | reference | 1.86 (1.61–2.15) | 3.89 (2.40–6.29) |
|  |  | Multivariable | reference | 1.35 (1.15–1.58) | 2.15 (1.28–3.61) |
|  | Male | Univariable | reference | 2.46 (1.88–3.21) | 8.48 (4.45–16.15) |
|  |  | Multivariable | reference | 1.76 (1.32–2.37) | 3.78 (1.96–7.26) |
|  | Female | Univariable | reference | 1.79 (1.51–2.13) | 2.38 (1.17–4.87) |
|  |  | Multivariable | reference | 1.19 (0.98–1.44) | 1.24 (0.60–2.56) |
|  |  |  | *P _Interaction_* | <0.001 | <0.001 |
| Pain/discomfort | Overall | Univariable | reference | 1.40 (1.26–1.56) | 2.06 (1.29–3.30) |
|  |  | Multivariable | reference | 1.22 (1.09–1.37) | 1.44 (0.88–2.37) |
|  | Male | Univariable | reference | 1.48 (1.23–1.77) | 3.36 (1.80–6.27) |
|  |  | Multivariable | reference | 1.30 (1.07–1.56) | 2.36 (1.28–4.37) |
|  | Female | Univariable | reference | 1.49 (1.30–1.70) | 1.48 (0.75–2.90) |
|  |  | Multivariable | reference | 1.21 (1.05–1.39) | 0.90 (0.48–1.68) |
|  |  |  | *P _Interaction_* | <0.001 | <0.001 |
| Anxiety/depression | Overall | Univariable | reference | 1.21 (1.04–1.41) | 1.24 (0.72–2.12) |
|  |  | Multivariable | reference | 1.15 (0.65–1.96) | 1.13 (0.65–1.96) |
|  | Male | Univariable | reference | 1.48 (1.14–1.92) | 1.94 (0.92–4.11) |
|  |  | Multivariable | reference | 1.33 (1.01–1.74) | 1.32 (0.60–2.90) |
|  | Female | Univariable | reference | 1.20 (0.99–1.45) | 1.06 (0.49–2.29) |
|  |  | Multivariable | reference | 1.08 (0.89–1.31) | 0.96 (0.45–2.02) |
|  |  |  | *P _Interaction_* | 0.004 | 0.083 |

Values are presented as coefficient (95% confidence interval) in total calories and odds ratio (95% confidence interval) in high-intensity physical activity and EQ-5D values. Multivariable analysis was adjusted for age (≥65 years versus <65 years), sex, smoking status (never smokers versus current- or ex-smokers), BMI (BMI ≥25 kg/m^2^ versus 18.5 kg/m^2^ ≤ BMI < 25 kg/m^2^ versus BMI <18 kg/m^2^ ), education level (high school or less versus college or above), family income (upper half versus lower half), and the number of comorbidities (one or less versus two or more). *P* for interaction (*P _Interaction_*) was obtained from likelihood ratio tests for interaction with normal spirometry/mild-to-moderate RSP/severe RSP and sex.

RSP, restrictive spirometric pattern; EQ-5D, EuroQoL five-dimension questionnaire; BMI, body mass index.
